# Ectopic Wnt/Beta–Catenin Signaling Induces Neurogenesis in the Spinal Cord and Hindbrain Floor Plate

**DOI:** 10.1371/journal.pone.0030266

**Published:** 2012-01-19

**Authors:** Milan Joksimovic, Meera Patel, Makoto Mark Taketo, Randy Johnson, Rajeshwar Awatramani

**Affiliations:** 1 Department of Neurology and Center for Genetic Medicine, Feinberg Medical School, Northwestern University, Chicago, Illinois, United States of America; 2 Department of Pharmacology, Graduate School of Medicine, Kyoto University, Yoshida-Konoé-cho, Sakyo, Kyoto, Japan; 3 Department of Biochemistry and Molecular Biology, University of Texas, M. D. Anderson Cancer Center, Houston, Texas, United States of America; Seattle Children's Research Institute, United States of America

## Abstract

The most ventral structure of the developing neural tube, the floor plate (FP), differs in neurogenic capacity along the neuraxis. The FP is largely non-neurogenic at the hindbrain and spinal cord levels, but generates large numbers of dopamine (mDA) neurons at the midbrain levels. Wnt1, and other Wnts are expressed in the ventral midbrain, and Wnt/beta catenin signaling can at least in part account for the difference in neurogenic capacity of the FP between midbrain and hindbrain levels. To further develop the hypothesis that canonical Wnt signaling promotes mDA specification and FP neurogenesis, we have generated a model wherein beta–catenin is conditionally stabilized throughout the FP. Here, we unambiguously show by fate mapping FP cells in this mutant, that the hindbrain and spinal cord FP are rendered highly neurogenic, producing large numbers of neurons. We reveal that a neurogenic hindbrain FP results in the altered settling pattern of neighboring precerebellar neuronal clusters. Moreover, in this mutant, mDA progenitor markers are induced throughout the rostrocaudal axis of the hindbrain FP, although TH+ mDA neurons are produced only in the rostral aspect of rhombomere (r)1. This is, at least in part, due to depressed Lmx1b levels by Wnt/beta catenin signaling; indeed, when Lmx1b levels are restored in this mutant, mDA are observed not only in rostral r1, but also at more caudal axial levels in the hindbrain, but not in the spinal cord. Taken together, these data elucidate both patterning and neurogenic functions of Wnt/beta catenin signaling in the FP, and thereby add to our understanding of the molecular logic of mDA specification and neurogenesis.

## Introduction

The floor plate (FP) has long been recognized as a structure at the ventral midline of the central nervous system (CNS), conspicuously negative for neuronal elements [1], [2]. Recent studies have confirmed that the FP does not generate large numbers of neurons in the spinal cord and hindbrain regions [3], [4]. In contrast, at midbrain levels the FP is remarkably neurogenic, generating among others, midbrain dopamine neurons (mDA; [3]–[12]).

The stark difference in neurogenic capacity is at least in part, due to canonical Wnt signaling. First, Wnt1 is expressed in the ventral midbrain but not ventral hindbrain or spinal cord [13]–[15] [16] and the Wnt1 null mutant has severely reduced numbers of mDA [15]. Next, both FP-specific, and temporally controlled conditional ablation of *beta–catenin* results in the reduction of mDA neurogenesis [4], [17], [18]. In these mutants, the midbrain FP, at least partially loses expression of Otx2, Msx1, Ngn2 [4] and Lmx1a [4], [18], maintains the expression of *Shh*, and thereby molecularly resembles the hindbrain FP [4]. These studies suggest that canonical Wnt signaling is necessary for mDA specification and neurogenesis, although it is possible that some of the effects seen in these studies are due to loss of integrity of adherens junctions [17]. In accordance with the notion that Wnt signaling is critical for mDA production, a recent study has used canonical Wnt-promoting regimens to greatly improve mDA derived from human embryonic stem cells [19].

To determine whether Wnt signaling is sufficient to induce mDA production, we generated *Shh::cre;Ctnnb1^lox(ex3)^* embryos in which beta–catenin was stabilized in the FP [4]. Analysis of the hindbrain FP in such embryos, revealed the induction of Lmx1a, Otx2, Msx1, and Ngn2, and suppression of *Shh* in the hindbrain. Since forced expression of Otx2 alone in the hindbrain FP is sufficient to generate mDA neurons [6], our expectation was that in the *Shh::cre;Ctnnb1^lox(ex3)^* embryos, induction of Otx2 would be sufficient to drive mDA production. However, TH+ mDA were not observed emanating from most regions of the Otx2+ hindbrain FP. This finding, coupled with the fact that the mutant FP, at later stages, lacked the marker Lmx1b (not shown), led us to postulate that ectopic mDA were not generated in this mutant in part, because of sustained Wnt/beta–catenin signaling [4]. Thus, although Wnt signaling could activate key mDA transcription factors in the hindbrain FP, genesis of mDA neurons was not achieved throughout most of the hindbrain.

Since Wnt/beta–catenin signaling is an integral component of the molecular logic of mDA genesis, we sought to extend our previous analyses on FP specific beta-catenin stabilization. Specifically, in *Shh::cre;Ctnnb1^lox(ex3)^* embryos, we wanted to a) determine the fate of hindbrain and spinal cord (SC) FP cells b) determine the consequences of a neurogenic FP on neighboring neuronal clusters c) determine why mDA were not generated through most of the hindbrain despite Lmx1a and Otx2 induction. To address these issues, first, we fate mapped FP cells in *Shh::cre;Ctnnb1^lox(ex3)^* embryos and revealed robust neuron production in the hindbrain and rostral SC. We also show that, at the hindbrain levels, the integrity of the septum medullae is disrupted which results in fusion of neuronal clusters that are normally bilaterally separated. With respect to patterning, mDA progenitor markers are induced throughout the rostrocaudal extent of the hindbrain. However, at all levels analyzed, except for the most rostral aspect of rhombomere (r)1, mDA were not generated from the FP. Instead, several ectopic neurons were characterized by a marker of the red nucleus, Brn3a, as well as Nkx6.1. We demonstrate that the lack of mDA is at least in part, a consequence of Lmx1b downregulation, caused by sustained beta–catenin signaling. Together, these studies extend previous observations on the patterning and neurogenic functions of Wnt signaling in the context of the FP and in cell-based mDA protocols [4], [15], [17], [19]–[22].

## Results

### Stabilization of beta–catenin in the hindbrain and SC FP results in neurogenesis

**Figure 1:**
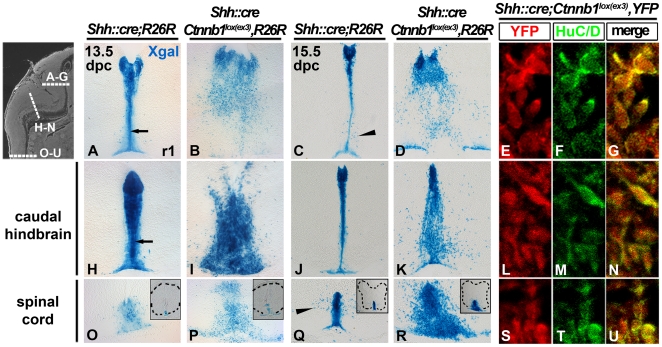
Ectopic Wnt/beta–catenin signaling in the hindbrain and spinal cord FP results in neuronal production. The hindbrain and spinal cord FP cells were lineage traced in control (*Shh::cre;R26R*; A, C, H, J, O, and Q) and mutant embryos (*Shh::cre;Ctnnb1^lox(ex3)^,R26R/YFP*; B, D, E–G, I, K, L–N, P, R, and S–U) at the rhombomere 1 (r1; A–G), caudal hindbrain (H–N), and spinal cord (O–U) levels using indicated antibodies (E–G, L–N, and S–U) and Xgal labeling (A–D, H–K, and O–R). Note that in mutants large numbers of Xgal+ cells are found in the parenchyma of the hindbrain and spinal cord; some staining of the end feet is still observed at the pial surface. Panels E–G, L–N, and S–U are high power confocal images with YFP as a lineage tracer. Newly generated neurons were visualized using HuC/D antibodies (green). In control hindbrain and spinal cord, an Xgal+ septal structure is observed (arrows in A and H); this Xgal+ septal structure persists till birth, but is not evident at P21. Some Xgal+ cells are detected in the parenchyma (arrowheads in C and Q). At these later stages several Xgal+ cells are observed in the hindbrain and spinal cord parenchyma, likely because of de novo Shh expression in these populations (not shown). Approximate levels of sections are shown in the DAPI labeled sagittal section in the upper left corner. Spinal cords are outlined with black dotted lines in the O–R insets.

We first aimed to determine the fate of FP cells expressing stabilized beta–catenin (Figures 1, S1). To do this, we generated *Shh::cre;Ctnnb1^lox(ex3)^* embryos harboring a R26R or R26YFP allele, and corresponding controls. In 13.5 and 15.5 dpc *Shh::cre;R26R/YFP* controls, Xgal is restricted to a small group of cells at the midline, which extend processes to the pial surface (Figure 1A, C, H, J, O, and Q; arrows in Figure 1A, H). In control hindbrain and spinal cord, this Xgal+ septal structure persists till birth, but is not evident at P21. Some Xgal+ cells are observed in the parenchyma (arrowheads in Figure 1C and Q); although few cells appear to be colabeled with HuC/D, many of these are not colabeled with TuJ1 or HuC/D suggestive of a glial lineage (not shown). In 13.5 and 15.5 dpc *Shh::cre;Ctnnb1^lox(ex3)^,R26R/YFP* mutant embryos, sections throughout the hindbrain and rostral SC reveal large numbers of Xgal+ cells in the mantle zone (1B, D, I, K, P, and R). Most of these labeled cells are colabeled with the early neuronal marker HuC/D (Figure 1E–G, L–N, and S–U). Thus, stabilized beta–catenin is sufficient to induce neurogenesis from the predominantly non-neurogenic hindbrain and SC FP.

### Hindbrain FP neurogenesis affects neighboring neuronal clusters

**Figure 2:**
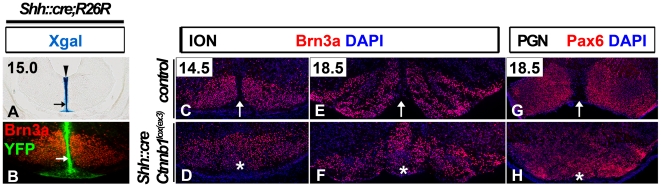
Bilateral separation of precerebellar nuclei is disrupted by the hindbrain floor plate stabilization of beta-catenin. (A) In *Shh::cre;R26R* embryos (15.0 dpc), Xgal labeling is detected in the hindbrain FP cells (arrowhead) which extend processes to the pial surface (arrow) forming the septum medullae. (B) Brn3a expression (red) is detected in two club-shaped clusters of the inferior olivary nucleus (ION) separated by the septum medullae labeled with YFP (green; arrow) in *Shh::cre;R26YFP* embryos (15.0 dpc). An approximate anterior-posterior and dorso-ventral level of the section shown in B corresponds to the region indicated by the arrow in A. (C–H) Coronal sections were labeled for Brn3a (ION marker; C–F) and Pax6 (pontine gray nucleus (PGN) marker; G and H) in control (C, E, and G) and *Shh::cre;Ctnnb1^lox(ex3)^* mutant embryos (D, F, and H) at indicated stages. Note that the two bilateral clusters of the ION and PGN nuclei normally separated by the septum medullae (arrows) appear to be fused at the midline in the mutants (asterisks).

We examined the consequences of ectopic hindbrain FP neurogenesis on ventral hindbrain nuclei. Normally, hindbrain FP cells extend processes to the pial surface (arrows in Figures 1A, H, and 2A, B) forming a structure largely devoid of neuronal elements, described as the septum medullae. In *Shh::cre;Ctnnb1^lox(ex3)^* mutants, the FP cells are neurogenic, and likely lose their attachment to the pial surface, thereby disrupting the integrity of the septum medullae. To determine the consequences of this disruption we examined settling of precerebellar neuron populations, which under normal circumstances, migrate from dorsal aspects of the neural tube and settle ventrally in bilateral clusters [23]. We observed that the inferior olivary nucleus (ION; Brn3a+/Foxa2−) and the pontine gray (PGN; Pax6+), both bilaterally symmetrical nuclei, appeared fused in the absence of the septum medullae (Figure 2D, F, and H). Thus, at least one deleterious consequence of a neurogenic hindbrain FP is the loss of a barrier that may allow bilateral separation of distinct hindbrain nuclei. Bilateral separation of these clusters may be critical for the coordination of movement of organisms with a bilaterally symmetrical body plan.

### Brn3a+ neurons and few mDA neurons are generated from the hindbrain FP in stabilized beta–catenin mutants

**Figure 3:**
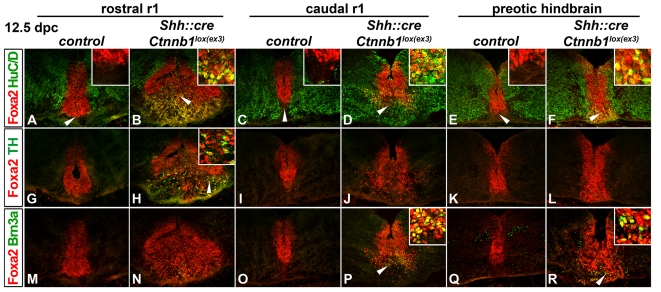
The hindbrain FP yields Brn3a+ and spatially restricted, mDA lineage neurons in *Shh::cre;Ctnnb1^lox(ex3)^* mutant embryos. 12.5 dpc horizontal (A–D, G–J, M–P) and coronal sections (E, F, K, L, Q, R) were labeled with indicated antibodies at the rostral r1 (A, B, G, H, M, N), caudal r1 (C, D, I, J, O, P), and preotic hindbrain levels (E, F, K, L, Q, R) in control (A, C, E, G, I, K, M, O, Q) and mutant embryos (B, D, F, H, J, L, N, P, R). Note that the Foxa2+/TH+ neurons are generated only at the rostral r1 levels; at this level, and to a lesser extent at caudal levels, the integrity of the FP appears to be disrupted (B, H, N). Ectopic Foxa2+/Brn3a+ neurons are produced predominantly in the more caudal hindbrain regions (P, R); in the most caudal hindbrain and rostral spinal cord regions neither Foxa2+/Brn3a+ nor Foxa2+/TH+ neurons are produced (not shown). Arrowheads depict the areas shown in the corresponding insets at the high magnification.

We next examined the types of neurons that are generated from the hindbrain FP in *Shh::cre;Ctnnb1^lox(ex3)^*embryos. Normally, in the midbrain, two key cell types are generated from Foxa2+/Shh+ progenitors; mDA, and a Brn3a+ cohort that populates the red nucleus, Edinger Westphal region (EW; not necessarily the preganglionic neurons) and supraoculomotor nucleus and cap (Su3) region. Additionally, Foxa2+/Nkx6.1+ neurons are also observed in distinct midbrain clusters (Joksimovic and Awatramani, unpublished observations; [24]). mDA are largely produced from midline Lmx1a/b+ progenitors while Brn3a+ neurons are predominantly generated from the lateral aspects of the *Shh*+ progenitor domain. Some mDA also appear to be produced from these lateral progenitors although the size of this cohort has not been determined [3]. We examined the production of these cell types in hindbrain regions of stabilized beta–catenin mutants. While most regions of the hindbrain were negative for mature mDA markers, the rostral most aspect of r1 revealed ectopic Foxa2+/TH+, Pitx3+/TH+, and Lmx1b+/TH+ mDA (Figures 3B, H, S2 and not shown) similar to observations in En1^Wnt1^ embryos [15]. 5-HT neurons are observed on either side of the medial TH+ neurons suggesting that this is bona fide hindbrain territory (Figure S2). Appearance of TH+ neurons in the hindbrain is likely not due to movement of mDA neurons from the midbrain to the hindbrain as the isthmic boundary, demarcated by Otx2, appears intact (Figure S3). In addition to mDA, in both rhombomere 1 and in more caudal hindbrain, Foxa2+/Brn3a+ neurons were ectopically generated from the FP (Figure 3P, R; note that these ectopic Brn3a+ neurons are distinct from the ION Brn3a+ neurons, in that they coexpress Foxa2). In addition to Foxa2+/Brn3a+ and Foxa2+/TH+ neurons, ectopic Foxa2+/Nkx6.1+ postmitotic neurons were also observed in the hindbrain (Figure S4). In the most caudal hindbrain (i.e. at the level of the ION) and rostral SC, neither Foxa2+/Brn3a nor Foxa2+/TH+ neurons were generated, although many YFP+/βgal+/Foxa2+/HuC/D+ cells were observed in these regions (Figures 1S–U, S1 and data not shown).

### Sustained beta–catenin signaling ultimately results in Lmxb gene downregulation and consequent lack of mDA

**Figure 4:**
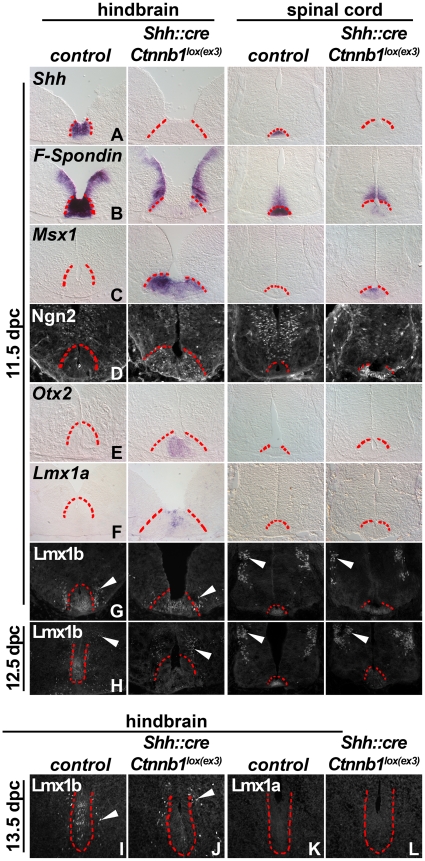
Wnt/beta–catenin signaling induces mDA progenitor genes but downregulates classical FP markers and Lmx1b. 11.5 (rows A–G), 12.5 (row H), and 13.5 dpc (I–L) hindbrain and spinal cord sections were labeled with indicated riboprobes (rows A–C, E, F) and antibodies (rows D, G, H, I–L) in control and *Shh::cre;Ctnnb1^lox(ex3)^* mutant embryos. Note that typical FP genes, *Shh* and *F-Spondin*, are downregulated at both axial levels. However, a set of upregulated genes includes *Msx1* and Ngn2 at both axial levels while *Otx2* and *Lmx1a* induction is restricted to the hindbrain levels. In contrast, Lmx1b is downregulated at both axial levels between 11.5–13.5 dpc. Although *Lmx1a* is initially induced (row F), its ectopic expression attenuates in the caudal to rostral direction at 12.5 dpc (not shown) and is undetectable by 13.5 dpc (L) in the hindbrain FP. Red dotted lines delineate the FP. Arrowheads depict Lmx1b+ serotonergic neurons and dI5 interneurons in the hindbrain and spinal cord, respectively.

We analyzed marker expression in the hindbrain and SC FP in stabilized beta–catenin mutants (Figure 4). The FP is conspicuously wide in the mutants, in part, a consequence of increased proliferation [4] and overall neurogenesis. Typical FP genes, *Shh* and *F-Spondin*, appeared to be downregulated at both axial levels (Fig. 4; rows A and B). In contrast, *Msx1*, Ngn2, *Otx2*, and *Lmx1a* were induced in the hindbrain FP, and of these *Msx1* and Ngn2 were induced at both axial levels (Figure 4; rows C–F) consistent with our previous observations [4]. Within the hindbrain FP, *Otx2* and *Lmx1a* were predominantly expressed medially, whereas *Msx1* and Ngn2 were induced in medial and lateral aspects of the FP. Despite the presence of mDA progenitor markers throughout the rostrocaudal extent of the hindbrain FP, in most regions, TH+ neurons were not observed emanating from the FP. In our earlier study, we speculated that persistence of stabilized beta–catenin in the FP cells could preclude their differentiation into mDA neurons [4].

Examination of 11.5–13.5 dpc embryos revealed a marked downregulation of Lmx1b in the hindbrain and SC FP in *Shh::cre;Ctnnb1^lox(ex3)^*embryos, compared to controls (Figure 4; rows G and H, I, J). In 13.5 dpc embryos, even ectopic expression of Lmx1a ceased in the hindbrain FP in the mutant embryos (Figure 4L). Thus, although beta–catenin initially is sufficient to induce *Lmx1a* in the hindbrain FP, sustained activity of beta–catenin ultimately, either directly or indirectly, results in marked downregulation of both Lmx1a and Lmx1b. Since Lmx genes are key mDA determinants, we reasoned that their downregulation could underlie the lack of mDA in the mutant hindbrain. This was supported by the finding that the FP in rostral r1 has slightly higher levels of Lmx1b compared to the remainder of the hindbrain FP (not shown) and that rostral r1 FP did give rise to mDA in the *Shh::cre;Ctnnb1^lox(ex3)^* mutants.

**Figure 5:**
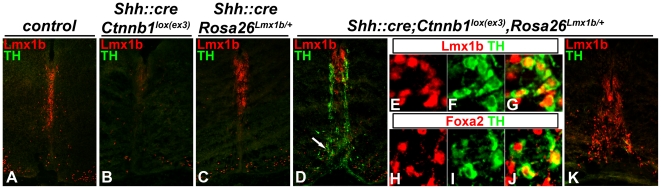
Sustained Wnt/beta–catenin signaling causes Lmx1b downregulation, which hampers the production of ectopic TH+ neurons. 13.5 dpc coronal sections were labeled for either Lmx1b/TH (A–G, K) or Foxa2/TH (H–J) at the otic (A–J) and postotic (K) hindbrain levels in control (A), *Shh::cre;Ctnnb1^lox(ex3)^* (B), *Shh::cre;Rosa26^Lmx1b/+^* (C) *and Shh::cre;Ctnnb1^lox(ex3)^,Rosa26^Lmx1b/+^* mutant embryos (D–K). Note the presence of ectopic TH+ neurons only upon forced expression of Lmx1b in the context of permanently stabilized beta–catenin in most of the hindbrain (D and not shown) but not at the postotic (inferior olivary nucleus) levels (K). Arrow in D indicates the area shown at the high magnification in E–G. H–J panels depict high magnification images of the section labeled for Foxa2 and TH adjacent to the section shown in D–G. Separate channel and merged images are shown in E–G, H–J.

If Lmx gene downregulation indeed underpinned the lack of mDA, then forced Lmx1b expression should overcome that limitation. To test this, we generated *Shh::cre;Ctnnb1^lox(ex3)^,Rosa26^Lmx1b/+^* embryos. In this scenario, Lmx1b expression is driven by a CAG promoter and therefore should be independent of Wnt/beta catenin mediated repression. In such embryos, Lmx1b expression is restored in the FP (Figure 5). Moreover, Lmx1b+/TH+ and Foxa2+/TH+ neurons are observed emanating from the FP in most of the rostrocaudal extent of the hindbrain (Figure 5D–J) until approximately the level of the inferior olivary nucleus, after which they were rarely detected (Figure 5K). These neurons were not detected in *Shh::cre;Ctnnb1^lox(ex3)^* or *Shh::cre;Rosa26^Lmx1b/+^* embryos at these axial levels (Fig. 5B and C). In fact, in *Shh::cre;Rosa26^Lmx1b/+^* embryos Lmx1a was not induced in most of the hindbrain FP, and neurogenesis was not observed. Taken together, these results suggest that although beta–catenin signaling is sufficient to induce mDA progenitor markers in the hindbrain FP, sustained beta–catenin signaling results in downregulation of *Lmx* genes, which at least in part, precludes mDA identity, but allows neurogenesis of other FP derived neuron types. If extrapolated to the Otx2+ midbrain, these data also suggest that in addition to their proposed role, in activating *Wnt1* in this region [4], [25], Lmx genes have a separate and essential role in establishing mDA neuron identity.

## Discussion

We use the hindbrain and spinal cord FP, structures normally scant in canonical Wnts, to study the capacity of Wnt/beta–catenin signaling. Our results shed light on two distinct functions of Wnt/beta catenin signaling – patterning and neurogenesis. We show that ectopic Wnt/beta–catenin signaling is sufficient to convert largely non-neurogenic structures, the hindbrain and SC FP, into highly neurogenic ones. Additionally, Wnt/beta catenin signaling can regulate the expression of key mDA progenitor specific genes – whereas Otx2, Msx1 and Ngn2 are induced, Lmx1b, Shh and F-spondin are decreased. Lmx1a is initially induced, but ultimately downregulated. Although Wnt/beta–catenin signaling induces mDA progenitor markers, ectopic mDA neurons are not observed in the most of the hindbrain except the rostral tip of r1. However, boosting Lmx1b levels increases the capacity of the hindbrain FP to generate mDA neurons.

The patterning and neurogenic functions of Wnt/beta–catenin signaling are spatially uncoupled. Gene cascades associated with neurogenesis, such as Msx1 and Ngn2 are altered both in the hindbrain and spinal cord FP. Genes associated with mDA patterning/specification such as Lmx1a and Otx2 are induced only in the hindbrain but not in the spinal cord FP. Further, within the hindbrain, these genes are induced only in the medial aspects of the FP. What might account for this spatial uncoupling of the patterning and neurogenic function of Wnt/beta–catenin signaling? First, it is possible that Wnt/beta–catenin signaling can only induce patterning genes at very early times. The medial hindbrain FP is recombined earlier than the spinal cord and lateral hindbrain FP by virtue of *Shh::cre* expression kinetics. Thus, it is feasible that these later recombined FP regions could have already passed a temporal window of sensitivity to Wnt/beta–catenin signaling and therefore become resistant to patterning alterations. A second possibility is that the hindbrain FP shares an embryonic origin with the midbrain FP, both being derived from a common group of cells [2]; these cells therefore are intrinsically similar, at least in the early embryo, and respond equivalently to Wnt/beta–catenin signaling by activating Lmx1a and Otx2. A third possibility is that hindbrain FP cells are exposed to a different milieu than spinal cord FP cells and therefore respond differently.

What does the spatial uncoupling of patterning and neurogenic functions of Wnt/beta–catenin signaling in the FP tell us in terms of hierarchical cascades in mDA specification? First, these data reveal that expression of Msx1 and Ngn2 as well as neuron production from the FP can occur in the absence of Lmx1a and Otx2. This is exemplified in the spinal cord in this mutant. The fact that Lmx1a/Otx2 are uncoupled from Msx1/Ngn2 suggests that neither Otx2 nor Lmx1a are required for Msx1/Ngn2 expression (this may not hold true for Lmx1b, which is present throughout the hindbrain and spinal cord FP). Taken together with other studies wherein Lmx genes activates Msx1 [7], the simplest model that fits all these data is that Lmx1a/b (initially only Lmx1b), in the Otx2 rich midbrain, activate Wnt1; Wnt/beta–catenin signaling is required for Lmx1a and Otx2 expression, but also activates Msx1/Ngn2, represses Shh, and promotes neurogenesis [4]. In accordance with this model, a recent chromatin immunoprecipitation study has indeed revealed a direct interaction of Lmx1a/b with Wnt1 regulatory elements [25].

Our data also demonstrate that Wnt signaling is sufficient to downregulate net levels of Lmx genes, particularly Lmx1b. Although initially Wnt signaling is necessary and sufficient for Lmx1a expression [4], [18], [25], Wnt signaling ultimately results in the downregulation of Lmx1a/b in the hindbrain FP. Indeed, this decrease in Lmx stoichiometry is, in part, the reason why mDA neurons are restricted to rostral r1, despite the induction of mDA markers throughout the hindbrain. Boosting Lmx1b levels, in the context of this mutant, was sufficient to at least partially facilitate mDA neurogenesis throughout most of the hindbrain, but not spinal cord. Extrapolating to the midbrain, these data also suggest that the timing and intensity of Wnt/beta–catenin signaling need to be tightly regulated to achieve the correct transcription factor stoichiometries. This is likely achieved by a balance of positive and negative regulators of canonical Wnts and their signaling components.

Our data, which for the first time show lineage traced hindbrain FP derived neurons, also demonstrate the unwanted consequence of hindbrain FP neurogenesis. We reveal that the hindbrain FP normally extends processes to the pial surface that allow bilateral separation of hindbrain nuclei. When the hindbrain FP is neurogenic, this septal structure is disrupted, and consequently at least two important populations, the inferior olivary and pontine gray nuclei, appear fused at the midline, rather than bilaterally symmetrical. The loss of bilateral separation of these key structures could have detrimental consequences on motor coordination for a bilaterally symmetrical organism. Perhaps for this reason, SFRPs are normally expressed in the hindbrain and SC FP (Joksimovic, M and Awatramani, R; unpublished observations), to antagonize the function of possible ventral canonical Wnt activity [26], [27].

In summary, we have demonstrated the potent proneurogenic role of Wnt/beta–catenin signaling in the FP. In the hindbrain, we show that consequent neuron production disrupts both the normal morphology of the septum medullae and adjacent neuron clusters. We also reveal that in addition to its ability to induce key mDA genes in the hindbrain FP, sustained Wnt/beta–catenin signaling downregulates a key mDA determinant – Lmx1b. Extrapolating to the midbrain, these data suggest that Wnt/beta–catenin signaling levels need to be carefully titrated for achieving the correct numbers and types of neurons for normal physiologic function.

## Materials and Methods

### Ethics statement

Mice were maintained and sacrificed according to the protocols approved by the Northwestern University Animal Care and Use Committee, protocol number 2008-1432.

### Mouse strains and genotyping

For fate mapping experiments, male mice bearing a *GFPcre* fusion that was knocked in to the *Shh* locus (hereafter designated *Shh::cre*; [28]) were crossed to *R26R* [29] or *R26YFP* female indicator mice to obtain *Shh:cre;R26R or Shh::cre;R26YFP* specimens. Mice harboring *Ctnnb1^lox(ex3)^* and *Rosa26^Lmx1b/+^* alleles have been previously described[30], [31]. For permanent stabilization of beta–catenin, female *Ctnnb1^lox(ex3)^* mice were crossed to *Shh::cre;R26R* or *Shh::cre;R26YFP* male mice to obtain *Shh::cre;Ctnnb1^lox(ex3)^,R26R* or *Shh::cre;Ctnnb1^lox(ex3)^,R26YFP* embryos. For rescue experiments, mice harboring *Lmx1b* conditional overexpression alleles (hereafter designed *Rosa26^Lmx1b/+^*) were crossed to *Ctnnb1^lox(ex3)^* mice to generate *Ctnnb1^lox(ex3)^*,*Rosa26^Lmx1b/+^* female mice. These mice were then crossed to *Shh::cre* male mice to obtain *Shh::cre;Rosa26^Lmx1b/+^* and *Shh::cre;Ctnnb1^lox(ex3)^,Rosa26^Lmx1b/+^* embryos. A morning of the day when a vaginal plug was detected was designated as 0.5 days postcoitum (dpc). For all panels presented, n>3.

### Xgal histochemistry, RNA in situ hybridization, and fluorescent immunohistochemistry

Embryos or whole brains were harvested and fixed in 0.2%–4% PFA in PBS for various amount of time depending upon embryonic ages, and sectioned at 20–30 µm. Detection of Xgal and *mRNA in situ* hybridization were performed as described [4]. For fluorescent immunohistochemistry, tissue sections were postfixed in 1%–4% PFA in PBS, rinsed in PBS, blocked in 5% donkey serum, 0.1% Triton X-100 in PBS, and incubated overnight at 4°C with primary antibodies diluted in blocking solution: goat βgal (Biogenesis; 1∶1,500), mouse HuC/D (Molecular Probes; 1∶100), rabbit GFP (Molecular probes; 1∶1,500), guinea pig Lmx1b (1∶10,000), rabbit Lmx1b (1∶5,000), rabbit and sheep TH (Pel Freeze; 1∶500 and 1∶250, respectively), goat Foxa2 (Santa Cruz; 1∶50), rabbit Brn3a (1∶4,000), mouse Brn3a (Santa Cruz; 1∶100), rabbit Pitx3 (Zymed; 1∶500), rabbit 5-HT (Sigma; 1∶500), mouse Pax6 (Developmental Studies Hybridoma Bank; 1∶50), rabbit Lmx1a (1∶1,000), guinea pig Lmx1a (1∶20,000), and mouse Nkx6.1 (Developmental Studies Hybridoma Bank; 1∶100). Sections were rinsed in PBS and incubated with appropriate Alexa 488, 555, and 647 (Molecular Probes) or Cy3 and Cy5 (Jackson ImmunoResearch) secondary antibodies diluted 1∶250 in blocking solution, rinsed in PBS, covered with DAPI (1 mg/mL; Sigma) in PBS, rinsed in PBS, and coverslipped followed by epifluorescent (Leica) or confocal microscopy (Zeiss LSM 510 META laser scanning). Images were processed in Adobe Photoshop CS2.

## Supporting Information

Figure S1 **Hindbrain and spinal cord FP neurogenesis in** ***Shh::cre;Ctnnb1^lox(ex3)^,R26R*** **mutant embryos.** Lineage tracing of the hindbrain and spinal cord floor plate cells in *Shh::cre;Ctnnb1^lox(ex3)^,R26R* mutant embryos at the rhombomere 1 (r1; A–C), preotic hindbrain (D–F), and spinal cord (G–I) levels using indicated antibodies. Note that the floor plate is highly neurogenic as a consequence of sustained Wnt/beta-catenin signaling. Approximate levels of sections are shown in the DAPI labeled sagittal section in the upper left corner. Arrows indicate the areas shown in the corresponding insets at the high magnification.
(TIF)

Figure S2 **Ectopic Wnt/beta-catenin signaling leads to a production of Pitx3+/TH+ neurons in the rostral r1 hindbrain.** Horizontal, rostral r1 sections were labeled for either Pitx3/TH (A–C) or 5-HT/TH (D) in control (A) and mutant embryos (B–D) at 12.5 (A and B) and 13.5 dpc (C and D). Note, the appearance of the 5-HT flanked ectopic TH+ neurons; in these, TH generally precedes the Pitx3 induction. Arrowhead indicates the area shown in the corresponding inset at high magnification.
(TIF)

Figure S3 **The isthmic boundary is intact in the** ***Shh::cre;Ctnnb1^lox(ex3)^*** **mutant embryos.** 11.5 dpc oblique coronal sections through the isthmus were labeled with the *Otx2* riboprobe in the control (A) and mutant embryos (B). Note that the position of the isthmus appears to be intact (arrowheads) while *Otx2* is ectopically induced in the hindbrain floor plate (arrow). hy, hypothalamus.
(TIF)

Figure S4 **The hindbrain FP yields Foxa2+/Nkx6.1+ neurons in** ***Shh::cre;Ctnnb1^lox(ex3)^*** **mutant embryos.** 12.0 dpc coronal sections were labeled with Foxa2 (red) and Nkx6.1 (green) antibodies at preotic hindbrain levels in control (A) and mutant embryos (B). Note the appearance of ectopic Foxa2+/Nkx6.1+ neurons (arrow) in the mutant.
(TIF)
