# Supplementary figures and images for: Ectopic Wnt/Beta–Catenin Signaling Induces Neurogenesis in the Spinal Cord and Hindbrain Floor Plate
Source: PLoS One. 2012 Jan 19;7(1):e30266. doi: 10.1371/journal.pone.0030266 (PMC3261891; doi:10.1371/journal.pone.0030266)

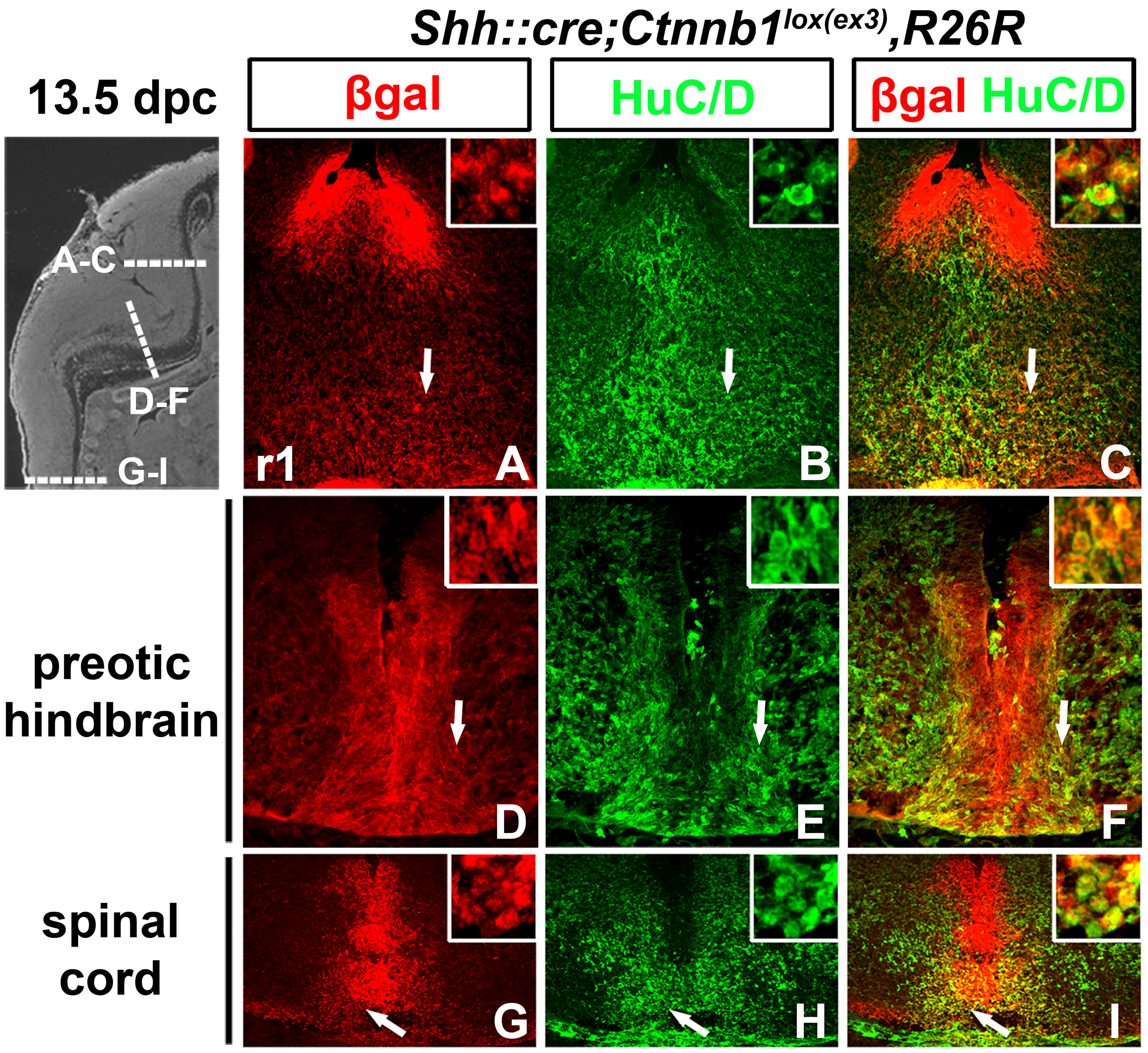

Supplement: Figure S1 — Hindbrain and spinal cord FP neurogenesis in Shh::cre;Ctnnb1lox(ex3),R26R mutant embryos. Lineage tracing of the hindbrain and spinal cord floor plate cells in Shh::cre;Ctnnb1lox(ex3),R26R mutant embryos at the rhombomere 1 (r1; A–C), preotic hindbrain (D–F), and spinal cord (G–I) levels using indicated antibodies. Note that the floor plate is highly neurogenic as a consequence of sustained Wnt/beta-catenin signaling. Approximate levels of sections are shown in the DAPI labeled sagittal section in the upper left corner. Arrows indicate the areas shown in the corresponding insets at the high magnification. (TIF) [file pone.0030266.s001.tif]

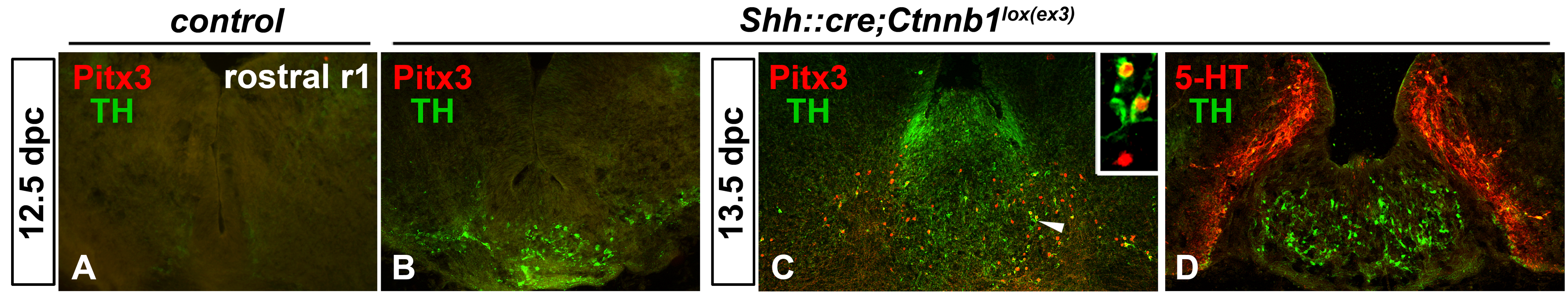

Supplement: Figure S2 — Ectopic Wnt/beta-catenin signaling leads to a production of Pitx3+/TH+ neurons in the rostral r1 hindbrain. Horizontal, rostral r1 sections were labeled for either Pitx3/TH (A–C) or 5-HT/TH (D) in control (A) and mutant embryos (B–D) at 12.5 (A and B) and 13.5 dpc (C and D). Note, the appearance of the 5-HT flanked ectopic TH+ neurons; in these, TH generally precedes the Pitx3 induction. Arrowhead indicates the area shown in the corresponding inset at high magnification. (TIF) [file pone.0030266.s002.tif]

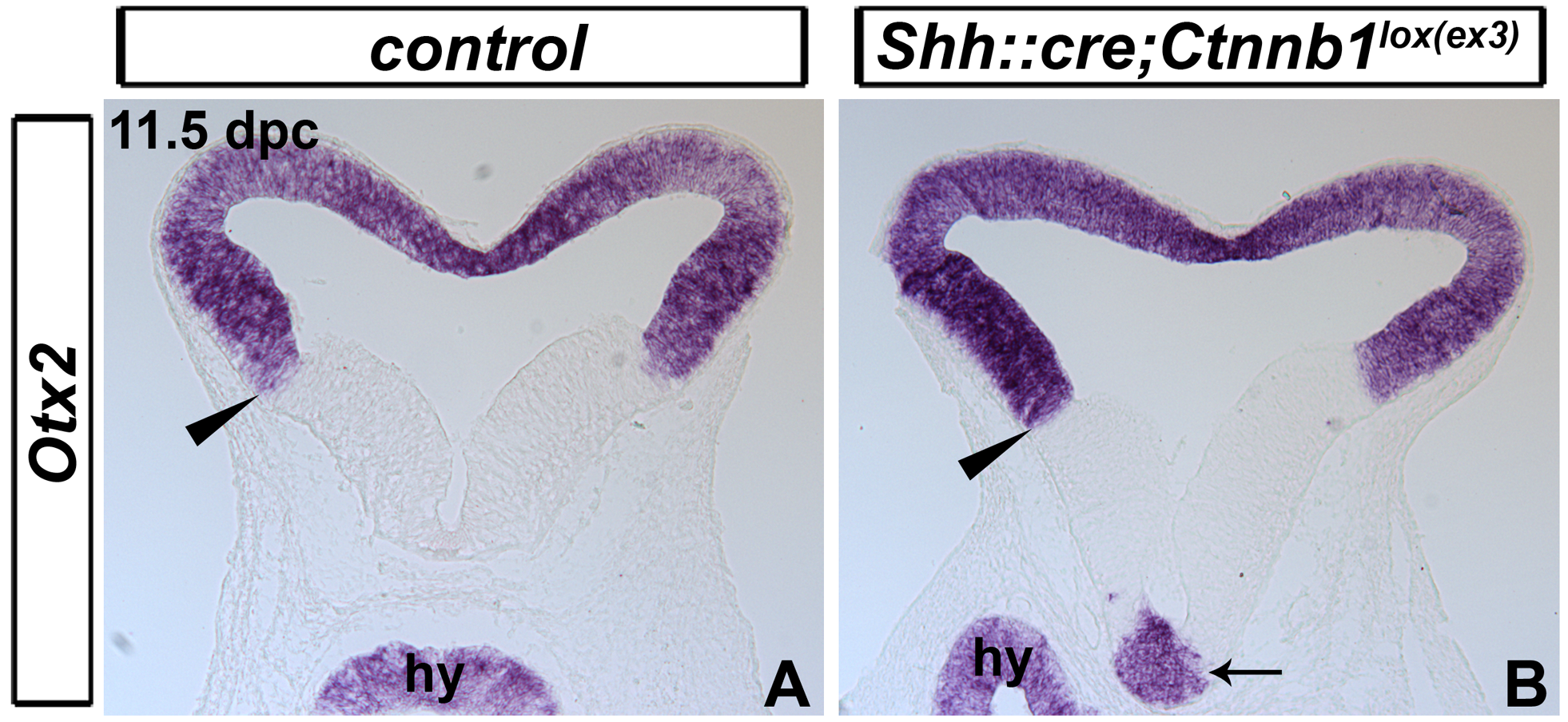

Supplement: Figure S3 — The isthmic boundary is intact in the Shh::cre;Ctnnb1lox(ex3) mutant embryos. 11.5 dpc oblique coronal sections through the isthmus were labeled with the Otx2 riboprobe in the control (A) and mutant embryos (B). Note that the position of the isthmus appears to be intact (arrowheads) while Otx2 is ectopically induced in the hindbrain floor plate (arrow). hy, hypothalamus. (TIF) [file pone.0030266.s003.tif]

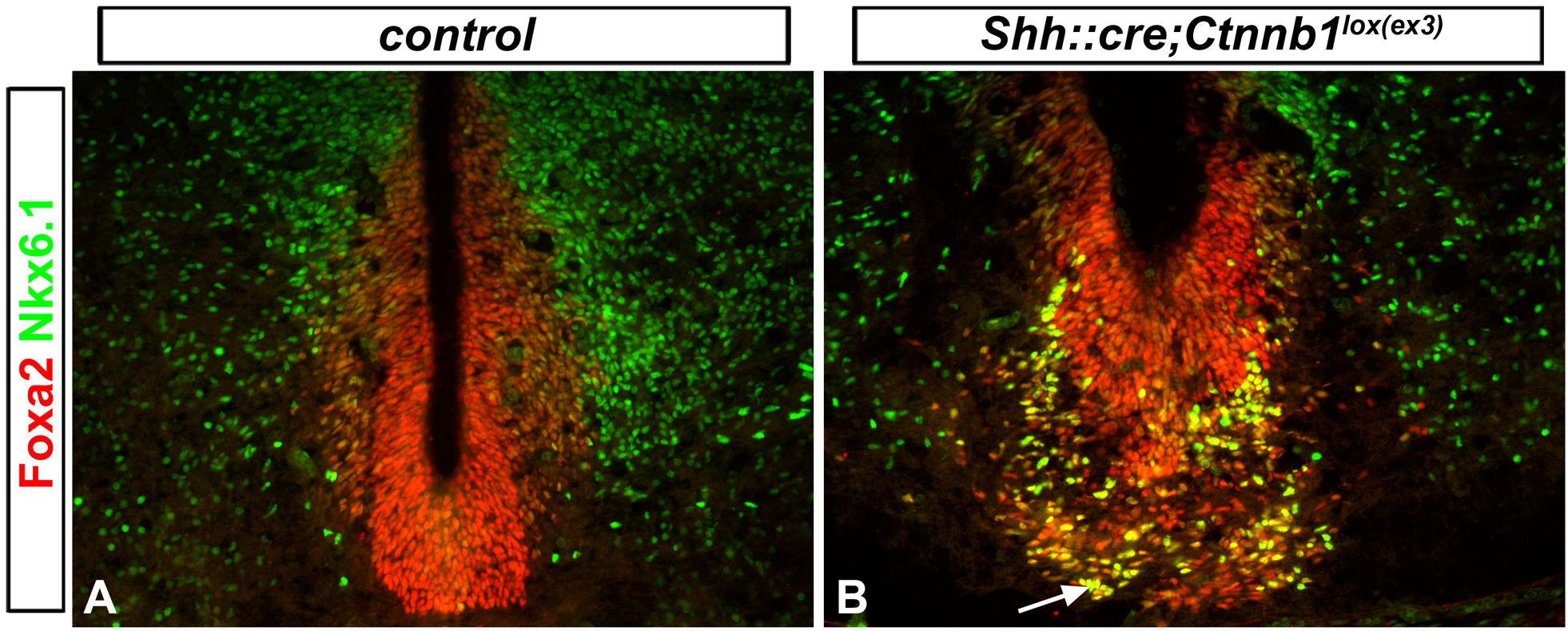

Supplement: Figure S4 — The hindbrain FP yields Foxa2+/Nkx6.1+ neurons in Shh::cre;Ctnnb1lox(ex3) mutant embryos. 12.0 dpc coronal sections were labeled with Foxa2 (red) and Nkx6.1 (green) antibodies at preotic hindbrain levels in control (A) and mutant embryos (B). Note the appearance of ectopic Foxa2+/Nkx6.1+ neurons (arrow) in the mutant. (TIF) [file pone.0030266.s004.tif]
